# Does leukocyte-platelet-rich fibrin (L-PRF) cause long term acceleration in the rate of canine retraction? A split-mouth, two-arm parallel group, randomized control trial

**DOI:** 10.1590/2177-6709.28.5.e232388.oar

**Published:** 2023-11-03

**Authors:** Seema GUPTA, Eenal BHAMBRI, Manish SHARMA, Mubasshir Ahmed SHAIKH, Amit ZOPE, Bhushan THOKE, Monika SOROKHAIBAM

**Affiliations:** 1 PM Dental College, Department of Orthodontics (Dhule, Maharashtra, India).; 2 Surendera Dental College and Research Institute, Department of Orthodontics (Sriganganagar, Rajasthan, India).; 3 ACPM Dental College, Department of Oral Pathology (Dhule, Maharashtra, India).

**Keywords:** L-PRF plugs, Platelet concentrate, Anchorage, Orthodontic treatment, Canine retraction

## Abstract

**Objective:**

The present study was conducted to investigate the effects of leukocyte-platelet-rich fibrin (L-PRF) on the rate of maxillary canine retraction for a period of 5 months.

**Methods:**

A split-mouth study was conducted on 16 subjects (9 males and 7 females; age range 17-25 years; mean age, 21.85±2.45 years) who required therapeutic extraction of bilateral maxillary first premolars. After the initial leveling and alignment, L-PRF plugs were placed in a randomly selected extraction socket (Experimental Group), and the other side served as a control (Control Group). Canine retraction was carried out by the activation of nickel-titanium (NiTi) closed-coil springs delivering 150 g of force. The rates of canine movement, canine rotation, tipping, root resorption, and molar movement were assessed at monthly intervals for five months (T0-T5). Pain, swelling and discomfort accompanying the procedure were assessed using a Likert scale.

**Results:**

The study revealed a significant increase in the rate of canine movement on the experimental side in the first two months, and significant molar anchorage loss was observed only in the first month for control side. There were no statistically significant differences between the groups regarding canine rotation, tipping, probing depth, root resorption, and pain perception.

**Conclusions:**

The use of L-PRF plugs in extraction sockets considerably enhanced the rate of canine movement only in the first two months, and long-term efficacy was not observed in this study.

## RESUMO

**Objetivo:**

O presente estudo foi realizado para investigar os efeitos da fibrina rica em leucócitos e plaquetas (L-PRF) na taxa de retração do canino superior, durante um período de cinco meses.

**Métodos:**

Um estudo de boca dividida foi realizado em 16 indivíduos (9 homens e 7 mulheres; faixa etária de 17 a 25 anos; idade média de 21,85 ± 2,45 anos) que precisavam de extração terapêutica dos primeiros pré-molares superiores de ambos os lados. Após o nivelamento e o alinhamento iniciais, os *plugs* de L-PRF foram colocados em um alvéolo pós-extração, selecionado aleatoriamente (Grupo Experimental), e o outro lado serviu como controle (Grupo Controle). A retração do canino foi realizada pela ativação de molas fechadas de níquel-titânio (NiTi) com 150 g de força. As taxas de movimentação do canino, rotação, inclinação e reabsorção radicular do canino e movimentação do molar foram avaliadas em intervalos mensais durante cinco meses (T0-T5). A dor, o inchaço e o desconforto após o procedimento foram avaliados por meio de uma escala de Likert.

**Resultados:**

O estudo revelou um aumento significativo na taxa de movimentação do canino no lado experimental nos dois primeiros meses, e uma perda significativa de ancoragem do molar foi observada apenas no primeiro mês no lado controle. Não houve diferenças estatisticamente significativas entre os grupos, com relação à percepção da dor e rotação, inclinação, profundidade de sondagem e reabsorção radicular do canino.

**Conclusões:**

O uso de *plugs* de L-PRF em alvéolos pós-extração aumentou consideravelmente a taxa de movimentação do canino apenas nos dois primeiros meses, não sendo observada uma eficácia em longo prazo.

## INTRODUCTION

The duration of orthodontic treatment is an important concern for adult patients who want their treatment to be completed as quickly as possible. Due to their busy schedules, they desire a shorter course and less chair-side time.^1^ Therefore, attempts have been made to accelerate orthodontic tooth movement (OTM). Various surgically-assisted procedures, such as corticotomy, piezocision, and micro-osteoperforation, have been used, but they are invasive in nature.^2^ ^,^ ^3^ On the other hand, non-surgical approaches^2^ show conflicting results regarding side effects such as systemic complications. 

One of the many strategies believed to be helpful in increasing OTM by enhancing the production of a variety of growth factors^4^ and effectively shortening the treatment duration is platelet-rich fibrin (PRF), which was first explored in France by Choukroun et al.^5^ Platelet concentrates are divided into two major categories depending on the presence of leukocytes and fibrin: platelet-rich plasma [Pure form (P-PRP), and leukocyte form (L-PRP)] and platelet-rich fibrin [pure form (P-PRF), and leukocyte-platelet-rich fibrin (L-PRF)].^6^ L-PRF has demonstrated more regular growth factor release from the delicate and flexible fibrin matrix, cost effectiveness, easier preparation, and longer effects than PRP.^7^

There are currently fewer human and animal studies on canine retraction using L-PRF plugs. Only four studies have been found in the literature on the effects of L-PRF plugs on OTM, and out of them, three studies were conducted for a period of two months.^8^ The disparities in the results of the studies may be due to the assessment techniques used to measure canine retraction. Two studies have used the distance between the distal marginal ridge of the canine and the mesial marginal ridge of the second premolar to assess the rate of canine movement. This led to confounding bias in the studies, as reduction of this distance might be due to anchorage loss, which was not assessed in the studies.^4^ ^,^ ^9^ The differences in the L-PRF preparation methods, platelet concentrates, and observation periods also led to the controversial results found in these studies. None of the studies evaluated anchorage loss and pain assessment in the patients. Therefore, the present study aimed to determine the effects of L-PRF on the rate of canine movement, anchorage loss, and pain perception, over a period of five months.

## MATERIAL AND METHODS

### STUDY DESIGN

**Figure 1:**
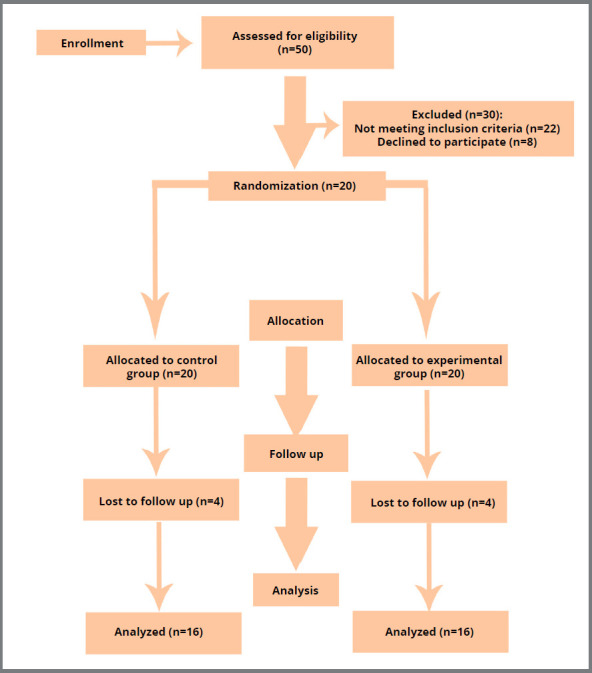
Consolidated Standards of Reporting Trials (CONSORT) flow diagram.

A single-center, two-arm, randomized control trial with split-mouth design was conducted on subjects recruited from the Department of Orthodontics at Surendera Dental College and Research Institute (January 2021-July 2021). The split-mouth design was chosen to avoid interpersonal variations in the study. Ethical clearance was obtained from the Institutional Ethical Committee (SDCRI/IEC/2020/012), and the trial was registered in the Clinical Trials Registry of India (CTRI) (REF/2022/02/051837). Informed consent was obtained from patients and/or legal guardians, prior to recruitment. The CONSORT statement was followed as a guide for this study (Fig 1). 

### SAMPLE SIZE CALCULATION

In this study, GPOWER statistical software (v. 3.1, Franz Faul, Universität Kiel, Kiel, Germany) was used to evaluate the sample size, assuming a mean difference and standard deviation (SD) of 0.55, type 1 error (α) of 0.05, and type 2 error (β) of 0.1 to achieve a statistical power of 90%^4^. The resultant sample size was 15, but considering sample attrition, it was decided to increase the sample size to 20. There was a sample attrition of 4 patients, due to changes in their residence; hence, the study was completed on 16 subjects. 

### RANDOMIZATION, ALLOCATION AND PATIENT SELECTION

A 1:1 allocation and simple randomization procedure of drawing lots by one investigator were used to allocate the side of the maxilla for placement of L-PRF plugs (Experimental Group, n=16), while the opposing side served as the split-mouth control, inducing secondary healing (Control Group; n=16). 

####  PICOS criteria

» Population: Class II division 1 or Class I bimaxillary protrusion patients requiring fixed mechanotherapy with first premolar extractions.
» Intervention: L-PRF plugs on the experimental side.
» Comparison: Control side with no L-PRF plug placement.
» Outcome: Primary outcome: Assessment of canine movement rate. Secondary outcome: rate of molar movement, assessment of canine angulation, root resorption, and pain perception.

Study design: Randomized controlled trial. 

The inclusion criteria were as follows: Subjects with Class II division 1 malocclusion or Class I bimaxillary protrusion, with relatively well-aligned arches, normodivergent growth pattern (FMA = 25 ± 5°), postpubertal as assessed by CVMI stage > 5, requiring therapeutic bilateral first premolar extractions with subsequent retraction of the canine, healthy oral and systemic conditions (probing depth <3 mm, plaque index <1 mm, no bleeding on probing), and no previous history of orthodontic treatment. Patients taking medications that could interfere with orthodontic tooth movement (NSAIDs, cortisone, hormones, anticoagulants), smoking, pregnancy, restorations, or endodontic treatments on maxillary canines, and patients with platelet disorders were excluded from the study.

### INTERVENTION

**Figure 2:**
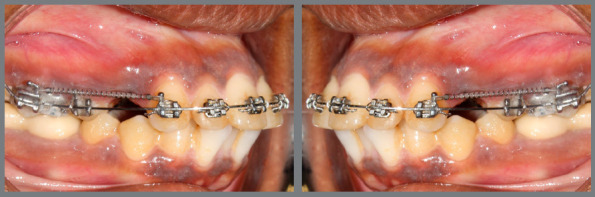
Retraction of maxillary canine with NiTi closed coil springs.

After obtaining complete pretreatment records and thorough oral prophylaxis, all patients started an orthodontic treatment procedure by one orthodontist, to prevent operator bias, using pre-adjusted Edgewise brackets (MBT 0.022-in slot). A soldered transpalatal arch (TPA), fabricated with 0.9mm stainless steel (SS) wire was used for anchorage reinforcement and maintenance of the transverse dimension. Leveling and alignment was started with a 0.014-in NiTi archwire, and completed when a 0.016×0.022-in SS archwire was placed for one month. Following, atraumatic extractions of maxillary first premolars and placement of L-PRF plugs were performed. NiTi closed-coil springs (Ormco^®^, Orange,California, USA) with a constant force of 150 g were used to retract the canines on both sides (Fig 2). 

####  L-PRF preparation and placement 

**Figure 3:**
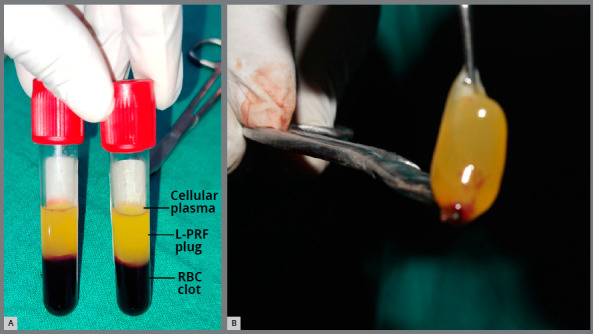
L-PRF preparation (A) and L-PRF plugs (B).

Using a 10-ml syringe, 9 ml whole venous blood sample was drawn quickly from the brachial vein and placed into two sterile tubes without the use of an anticoagulant (20-22 seconds on average). These tubes were then immediately centrifuged (in less than a minute) at 2,700 rpm (about 400-g centrifugation force, based on our estimations) for 12 minutes^10^ in IntraSpin centrifuge system (Intra-Lock International Boca, Raton, Fla). This caused a three-layer structure to form, with red blood cells at the bottom, cellular plasma with a straw color at the top, and platelets and a fibrin clot in the middle (Fig 3A). The middle portion (L-PRF) was collected, 2mm below the lower dividing line, after the upper straw-colored layer was removed, L-PRF plugs (Fig 3B) were placed in the socket using sterile tweezers, and compressed with amalgam condenser. The sockets were sutured using 4-0 vicryl sutures. Adhering to the protocols, with proper management of time period, is very critical and therefore, were carefully followed in the present study, to prevent dose- or procedure-dependent errors.^10^

### OUTCOME ASSESSMENT

All the required records, such as orthopantomograms (OPGs), intraoral periapical radiographs (IOPARs), and probing depths for maxillary canines, were taken prior to retraction (T0) and after 5 months (T5). Questionnaires for pain assessment were given to each patient to complete at home and return on the next appointment. Patients were discouraged from taking painkillers and when they did, in the event of severe pain, they were advised to write it down. Patients were recalled at intervals of 21 days for 5 months (T1-T5). Measurements were made on dental casts according to the procedure described in a previous study^11^, and presented bellow.

### MEASURING PROCEDURE

####  Evaluation of canine and first molar anteroposterior movement 

**Figure 4:**
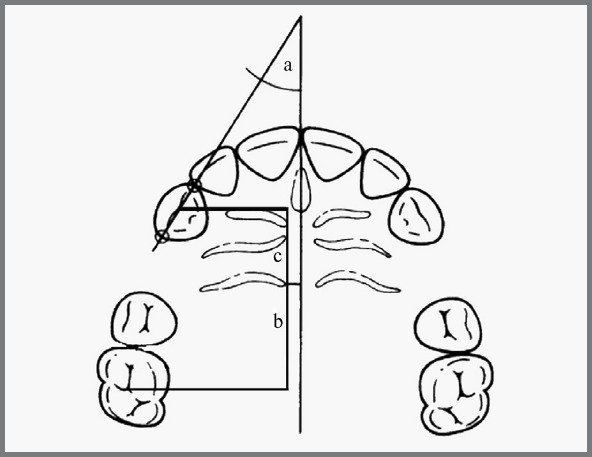
Schematic diagram depicting measurement of canine rotation (a), canine movement (b) and molar movement (c).

The amount of canine and molar movement, and of canine rotation were assessed using the method described by Zeigler and Ingervall^12^ (Fig 4). Photographs of the study models were taken by placing them vertically on a glass plate at a distance of 30 cm from the lens of the digital camera, as described by Azevedo et al.^13^ Before taking the photographs of the study model, the cusp tip of the canine, median palatal raphae, and third rugae were marked with a pencil. A perpendicular projection of the cusp tip of the canine and the central fossae of the first molar was drawn on the median line. The distance was measured from the medial rugae point of the third palatal rugae to assess movements of the canine and first molar monthly, for five months. All measurements were made using sliding digital calipers to the nearest 0.1 mm. Angles were measured using a protractor to the nearest 0.5°. 

####  Evaluation of root resorption and periodontal health of the canine 

Root resorption of the maxillary canine was assessed at T0 and T5 using IOPARs, taking index scores from 0-4, as described by Levander and Malmgren.^14^ Probing depth and attachment loss were assessed using an UNC #15 periodontal probe.

####  Evaluation of rotation and mesiodistal tipping of canine 

On the standardized photographs of the study models, the angle between the median raphae and the line through the mesial and distal edges of the canine was measured to assess canine rotation (Fig 4). Canine angulation was assessed using an OPG with a line through the orbital plane as a reference plane, as described by Ursi et al.^15^

####  Assessment of patient’s perception of pain, discomfort and satisfaction towards the procedure 

Following surgery, the patients received a questionnaire to complete, in order to determine their level of pain and discomfort. The questionnaire consisted of six items, five of which used a Likert scale with a four-point response range, and one with a three-point range. Participants were questioned about their subjective experiences with discomfort while eating, pain, and their perception of swelling on the surgical side at two different time points, T1 and T2, where T1 was 24 h following the surgical procedure and T2 was three days later.

### ERROR OF METHOD

To determine the errors associated with measurements, the measurements were repeated two weeks apart, by the same investigator, on 10 subjects. The intraclass correlation coefficient using Dahlberg formula, and paired *t*-tests were used to assess random and systematic errors in the study, respectively. 

### BLINDING

This was a single-blind study, in which the statistician was blinded with regard to the origin and grouping of data.

### STATISTICAL ANALYSIS

The measurements were analyzed statistically using SPSS v. 23 software (SPSS for Windows, release 7.51 Chicago, USA). The Shapiro-Wilk normality test was applied to assess the normality of the data. All variables except pain were found to be normally distributed; therefore, independent *t*-test was used to compare the mean differences of the two groups for monthly and overall canine and molar movements, as well as overall changes in canine rotation, angulation, root resorption and probing depths at the end of five months. The chi-square test was applied to compare the pain, swelling and discomfort scores across the two groups. Statistical significance was set at *p*≤ 0.05.

## RESULTS

The moderate to high reliability was observed with intraclass correlation coefficients between 0.88- 0.93 for all measurements. No statistically significant differences were found between the repeated measurements for any variable. The mean age of the subjects at the start of the treatment was 21.85±2.45 years. The rates of canine movement, molar movement, canine tipping and rotations, probing depth of canines, and root resorption of the canines were assessed at an interval of 21 days from T1 to T5 in 16 orthodontic patients. 

### PRIMARY OUTCOME

**Figure 5:**
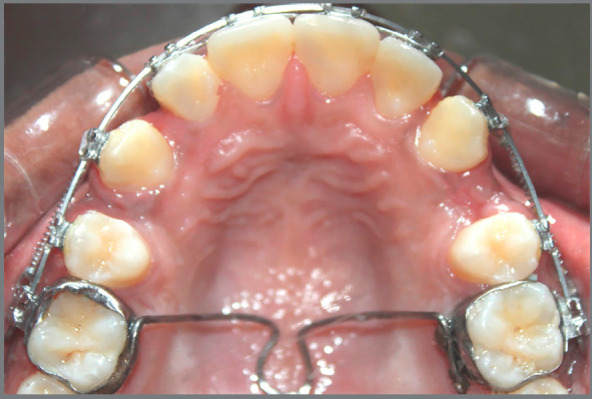
Difference in tooth movement of maxillary canine on experimental (right) and control (left) sides at T2.

There was a statistically significant greater rate of canine movement in the first two months (T0-T1 and T1-T2) in the experimental group [1.806 ± 0.404mm in the first month; 2.184 ± 0.297mm in the second month] than in the control group [1.294 ± 0.297mm in the first month; 1.875 ± 0.331mm in the second month]. The difference in the tooth movements on both sides is depicted in Figure 5. The total amount of canine retraction was 6.407 ± 0.336 mm on the experimental side and 5.546 ± 0.663 mm on the control side, and the difference between both was statistically significant (Table 1).

**Table 1:** Comparison of the rate of canine movement (mm) between the experimental and control groups.

Rate of canine retraction	Experimental Group Mean ± SD	Control Group Mean ± SD	T Value	P value	Significance
T0-T1	1.806 ± 0.404	1.294 ± 0.297	4.084	0.000*	Sig
T1-T2	2.184 ± 0.297	1.875 ± 0.331	2.779	0.009*	Sig
T2-T3	1.147 ± 0.442	0.906 ± 0.441	1.543	0.133	NS
T3-T4	0.531 ± 0.329	0.528 ± 0.413	0.022	0.982	NS
T4-T5	0.739 ± 0.303	0.943 ± 0.424	-1.566	0.128	NS
TOTAL	6.407 ± 0.336	5.546 ± 0.663	4.633	0.000*	Sig

SD = Standard Deviation; NS = Not Significant; * significant at p ≤ 0.05.

### SECONDARY OUTCOME

On the comparison of both groups, it was noticed that control group displayed more anchorage loss (0.473 ± 0.0705 mm), compared to experimental group (0.407 ± 0 .0719 mm), which was statistically significant only in the first month of treatment (T0-T1) and thereafter; being non-significant for the remaining time period (p ≥ 0.05) (Table 2).

**Table 2:** Comparison of molar movement (mm) rate between the experimental and control groups.

Rate of molar movement (mm)	Experimental Group Mean ± SD	Control Group Mean ± SD	T value	p value	Significance
T0-T1	0.407 ± 0 .0719	0.473 ± 0.0705	-2.6217	0.0136*	Sig
T1-T2	0.306 ± 0.0609	0.321 ± 0.0802	-0.5496	0.5797	NS
T2-T3	0.359 ± 0.2017	0.399 ± 0.2334	1.0318	0.4660	NS
T3-T4	0.500 ± 0.1204	0.486 ± 0.1250	-2.4048	0.3531	NS
T4-T5	0.239 ± 0.3371	0.243 ± 0.3230	0.3733	0.6613	NS
TOTAL	1.811 ± 0.3819	1.922 ± 0.3824	-0.8216	0.4178	NS

SD = Standard Deviation. NS = Not Significant. * Significant at p ≤ 0.05.

The study revealed non-significant changes in the amount of root resorption, mean probing depth, amount of canine rotation, and tipping in both groups during canine retraction (p ≥0.05) (Tables 3, 4).

**Table 3:** Comparison of the canine root resorption index and mean probing depths between the experimental and control groups.

	Root resorption index		p value	Probing Depth		p value
	Experimental Group Mean ± SD	Control Group Mean ± SD		Experimental Group Mean ± SD	Control Group Mean ± SD	
PRE	1.25 ± 0.433	1.188 ± 0.390	0.681	2.219±0.655	2.328 ± 0.629	0.37
POST	1.375 ± 0.484	1.313 ± 0.464	0.721	2.234±0.664	2.375 ± 0.619	2.17

SD = Standard Deviation. Significant at p ≤ 0.05.

**Table 4:** Comparison of the mean differences in the canine rotation and canine tipping between the experimental and control groups.

Canine rotation (degrees)		p value	Canine Tipping (degrees)		p value
Experimental Group Mean ± SD	Control Group Mean ± SD		Experimental Group Mean ± SD	Control Group Mean ± SD	
5.063 ± 3.614	4.938 ± 3.733	0.926	8.969 ± 0.760	9.000 ± 2.179	0.959

SD = Standard Deviation. Significant at p ≤ 0.05.

Assessment of pain, swelling, and discomfort following surgical intervention and the overall perception of discomfort showed non-significant differences. None of the patients reported using analgesics (Table 5).

**Table 5:** Assessment of pain, swelling, and discomfort following surgical intervention, using a four-point Likert scale, and overall perception of discomfort for the experimental and control groups.

Question	Group	Time	Scores				Chi value	P value	Significance
			1	2	3	4			
Q1	Experimental	T1	5	9	2	0	4.3	0.222	NS
	Control		3	6	5	2			
	Experimental	T2	3	7	5	1	1.57	0.664	NS
	Control		5	6	5	0			
Q2	Experimental	T1	9	4	3	0	5.38	0.06	NS
	Control		3	5	8	0			
	Experimental	T2	3	7	5	1	0.17	0.981	NS
	Control		3	8	4	1			
Q3	Experimental	T1	4	8	4	0	0.88	0.641	NS
	Control		4	10	2	0			
	Experimental	T2	9	6	1	0	0.54	0.765	NS
	Control		8	7	1	0			
Q4	Experimental	T1	4	9	3	0	0.54	0.76	NS
	Control		3	11	2	0			
	Experimental	T2	8	7	1	0	0.133	0.98	NS
	Control		7	8	1	0			
Q5	Experimental	T1	4	8	4	0	2.39	0.301	NS
	Control		4	10	2	0			
	Experimental	T2	4	8	4	0	0.376	0.828	NS
	Control		4	10	2	0			
Q6	Experimental	T1	4	8	4	0	1.47	0.478	NS
	Control		4	10	2	0			
	Experimental	T2	4	8	4	0	3.31	0.067	NS
	Control		4	10	2	0			

SD = Standard Deviation. NS = Not Significant. Significant at p ≤ 0.05.

## DISCUSSION

Due to the lack of sufficient data on the long-term influence of L-PRF on the rate of canine movement,^7^ the present study was conducted to determine the effects of L-PRF on the rate of canine movement. Most of studies on platelet concentrates were conducted using PRP and injectable-PRF (i-PRF). PRP has the highest autologous platelet concentration in a small amount of plasma. Different doses of PRP promote orthodontic tooth movement, according to animal studies.^8^ Submucosal injection of PRP or i-PRF have disadvantages related to pain, discomfort and swelling of the mucosa after injection, as well as the possibility of leakage during injection.^8^ ^,^ ^16^ In contrast, L-PRF has the advantages of simpler preparation and prolonged effects.^17^ ^,^ ^18^ In the present study, L-PRF plugs were used, as they behave as true fibrin tissue, maintain their fibrin structure even after seven days of placement, and slowly release more growth factors, mainly TGF-β, which has been shown to have anti-inflammatory properties, stimulate neoangiogenesis, increase proliferation of osteoblasts, and collagen synthesis, which triggers bone regeneration and accelerates tooth movement. Additionally, protease enzymes, other growth factors such as vascular endothelial growth factor (VEGF); platelet derived growth factor AB (PDGF AB), and matrix proteins such as fibronectin are also released by L-PRF plugs.^19^ As stated in PRP studies, the effects of L-PRF can also be related to the timing of release, concentration, and content of its growth factors. As the preparation of L-PRF plugs is very technique-sensitive and their acceleration effects are directly related to their dosage and method of preparation, a precise method, as suggested by Dohan et al,^10^ was used in the present study. After coming into contact with the glass, the blood sample without an anticoagulant almost instantly began to coagulate, which cut down on the amount of time needed to centrifuge fibrinogen. To get therapeutically useful L-PRF plugs charged with serum and platelets, the proper preparation technique must be followed, and quick handling is essential.

Immediately after careful extraction of first premolars, L-PRF plugs were inserted into the extraction socket to trigger regional acceleratory phenomenon (RAP). The experimental side of the trial, where the L-PRF plugs were inserted, displayed a higher rate of canine retraction than the control side, which was temporary only for the first two months. The findings of the present study are in accordance with previous studies.^20^ ^,^ ^21^ The 150-g of retraction force from the NiTi closed coil springs led to an increased rate of tooth movement even in the control group, which was in agreement with studies conducted by Bokas and Woods^22^ (1.85 mm) and Khanmasjedi et al^23^ (1.67 ± 0.39 mm). This was mainly a tipping movement, which was also noticed by Reyes Pacheco et al^24^ in their study, and might be due to constant and continuous force delivery by NiTi springs. The acceleration of canine movement was seen more in the second month, compared to the first month, which was similar to previous studies.^4^ ^,^ ^19^ ^,^ ^25^ This may be due to the slow release of BMP2 and TGF-β after a period of 7 days. The original L-PRF clots remained in good shape for a longer period of time.^10^ However, the present findings were in disagreement with the findings of Reyes Pacheco et al^24^ and Zeitounlouian et al.^25^ Reyes Pacheco et al^24^ found a decreased rate of retraction in 15 out of 17 patients. The disparity in the results might be due to the fact that they conducted the study on adults with a mean age of 33 years, who presented less periodontal response, compared to young adults in the present study.^26^ Moreover, they used the maxillary dental midline as reference landmark for assessment of canine movement, which itself is not a stable landmark, and this would be highly influenced by forces acting on the entire arch. They prepared L-PRF at 2700rpm for 14 min in 10ml of blood, and did not use the standardized procedure for preparing L-PRF; Xpression Box was also not used for compression of plug, which could have led to the methodological errors in the study, affecting the results.^10^ Zeitounlouian et al^25^ also did not find any acceleration effects of PRF in their study. They used i-PRF in their study and these conflicting results may be related to the different centrifugation protocols and methodology (700 rpm for 3 min). The centrifuge characteristics have a direct impact on the architecture and cell content of L-PRF clots. The different centrifugation speeds could result in a considerable flaw in all PRP/PRF studies.^10^ Other studies^4^ ^,^ ^9^ ^,^ ^27^ have found accelerated tooth movement in the L-PRF group, compared to the control group, at all time intervals. However, two^4^ ^,^ ^9^ of these studies were conducted for a period of 2-3 months and also had confounding bias in the method of assessment of canine movement, which might have affected the outcome. 

In the present study, all measurements were taken from the third palatal rugae, which is considered as stable structure.^28^ The reason for the short-term increase in OTM might be due to the short-term increase in the number of cells and production of cytokines, enhancing bone remodeling immediately following PRF application.^17^ However, the actual effects and mechanism of PRF need to be elucidated in further well-designed studies using standard protocols. Similar to PRP, L-PRF can also have dose-dependent effects; therefore, it is highly recommended to determine the concentrations of platelets and leukocytes in whole blood and L-PRF samples using ELISA before their application. 

During tooth movement, force application away from the center of resistance results in unwanted tipping and rotation. There were no appreciable differences between groups regarding canine rotation or inclination. These findings are consistent with those from other studies.^25^ ^,^ ^29^ ^,^ ^30^ ^,^ ^31^ On the other hand, Reyes Pacheco et al^24^ showed a larger canine rotation on the control side than on the experimental side. These conflicting results may be due to differences in methodology and platelet concentration. Comparing the probing depth and root resorption before and after the investigation, there were no discernible differences between the experimental and control groups. This is in accordance with previous studies.^21^ ^,^ ^22^ Assessment of perception of pain, swelling and discomfort showed non-significant results in the present study, corroborating the findings of previous studies.^5^ ^,^ ^32^ ^,^ ^33^ This was due to the anti-inflammatory effects of PRF.^34^ The primary limitation of this study was lack of evaluation of gender differences. 

### CLINICAL IMPLICATIONS

The results of this study support the short-term acceleration and anti-inflammatory effects of L-PRF on the rate of canine retraction, with less anchorage loss and no deleterious effects on the periodontium. However, long-term effects on the acceleration of canine retraction were not observed in this study; therefore, based on the findings of this study, further well designed randomized control studies in this regard are recommended. 

## CONCLUSION

The movement of the canines and molars, tipping, rotation, anchorage loss, and probing depth were evaluated, and it was concluded that the rate of canine retraction was statistically greater on the experimental side only in the first two months with the use of L-PRF. Anchorage loss was greater in the control group only in the first month of treatment. Canine tipping, rotation, root resorption, probing depth, and pain perception were statistically insignificant in both groups. 
